# Supplementary material for: Inactivation of the High-Affinity Ca2+ Uptake System Delays the Amiodarone-Induced Ca2+ Influx in Yeast Ogataea parapolymorpha
Source: Int J Mol Sci. 2025 Nov 25;26(23):11386. doi: 10.3390/ijms262311386 (PMC12692672; doi:10.3390/ijms262311386)
Supplement: Supplementary file 1 [file ijms-26-11386-s001.zip › ijms-3986000-supplementary.pdf]

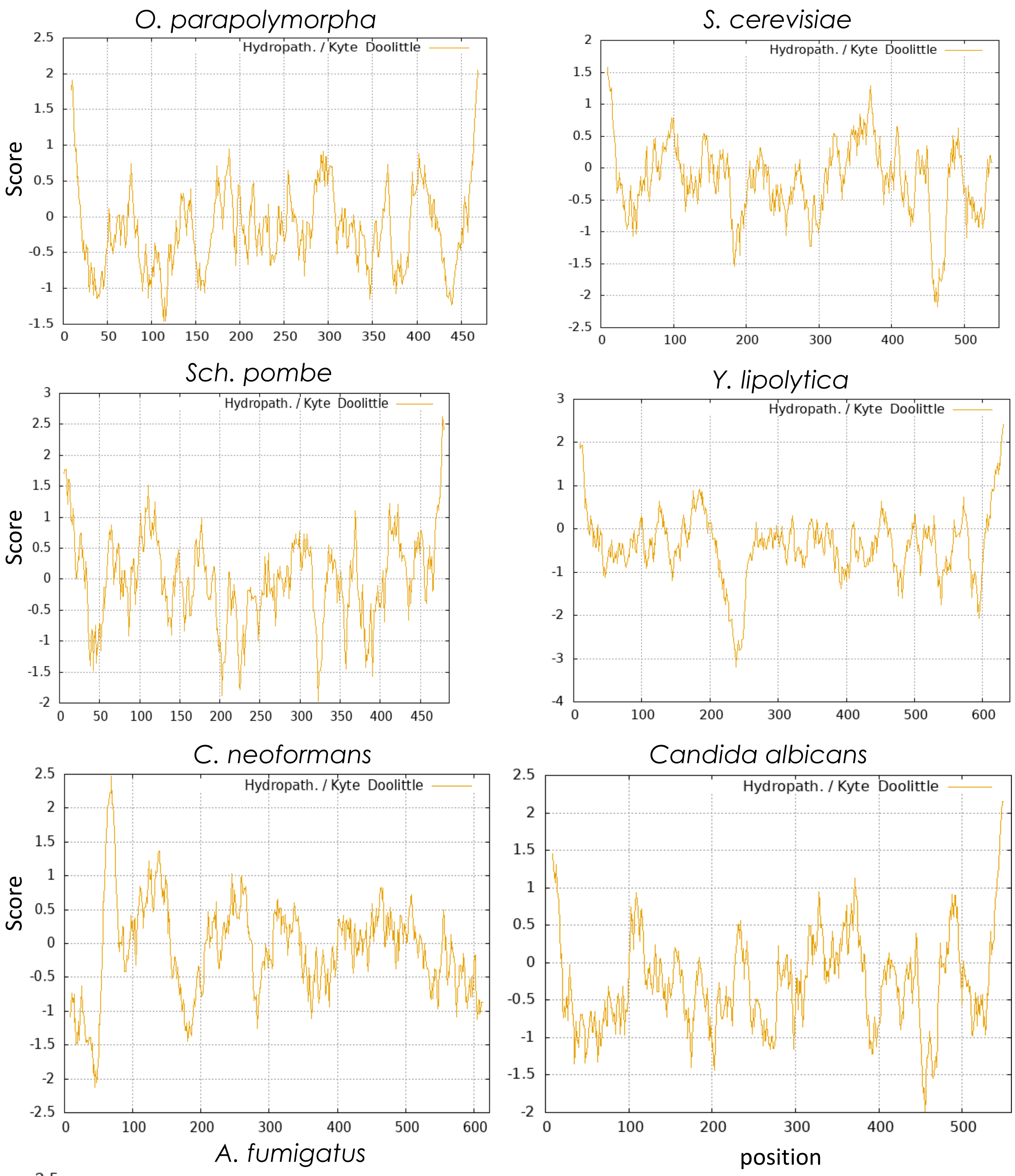

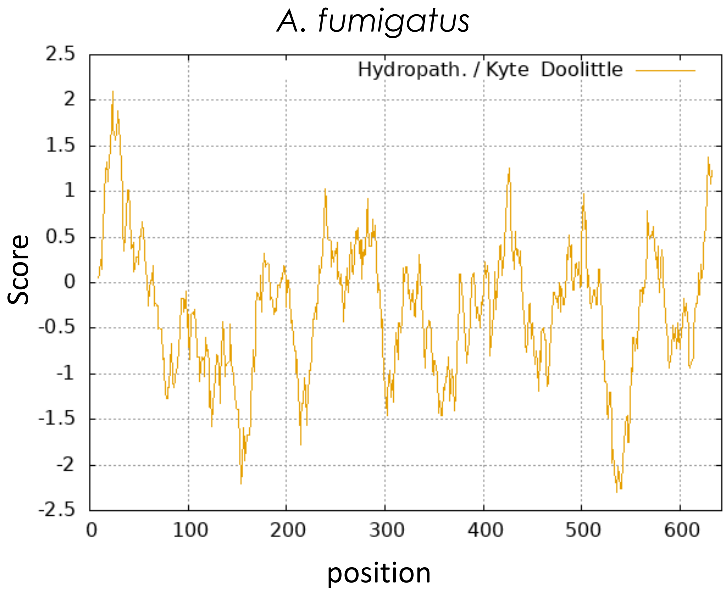

Figure S1. Hydropathy profiles of Mid1 homologs from different fungi. Window size =19 aa.

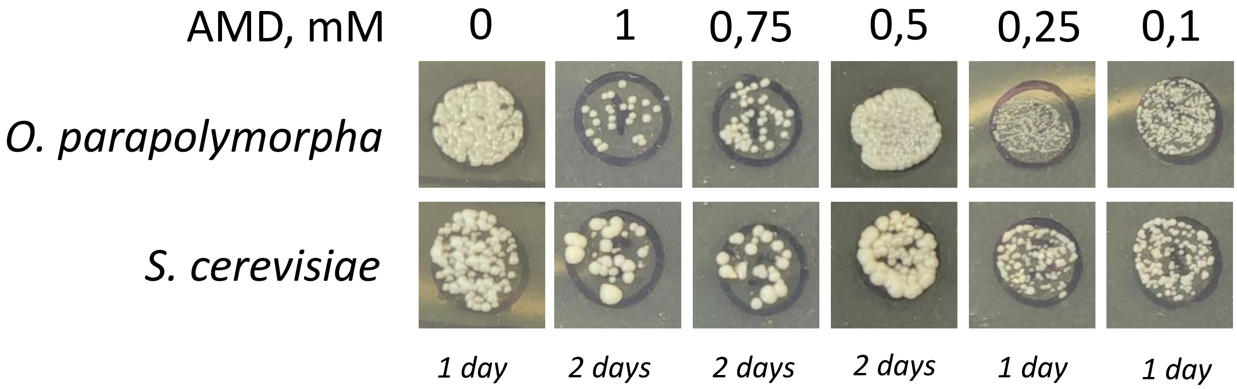

Figure S2. Growth of yeast colonies on medium containing different concentration of amiodarone (AMD).

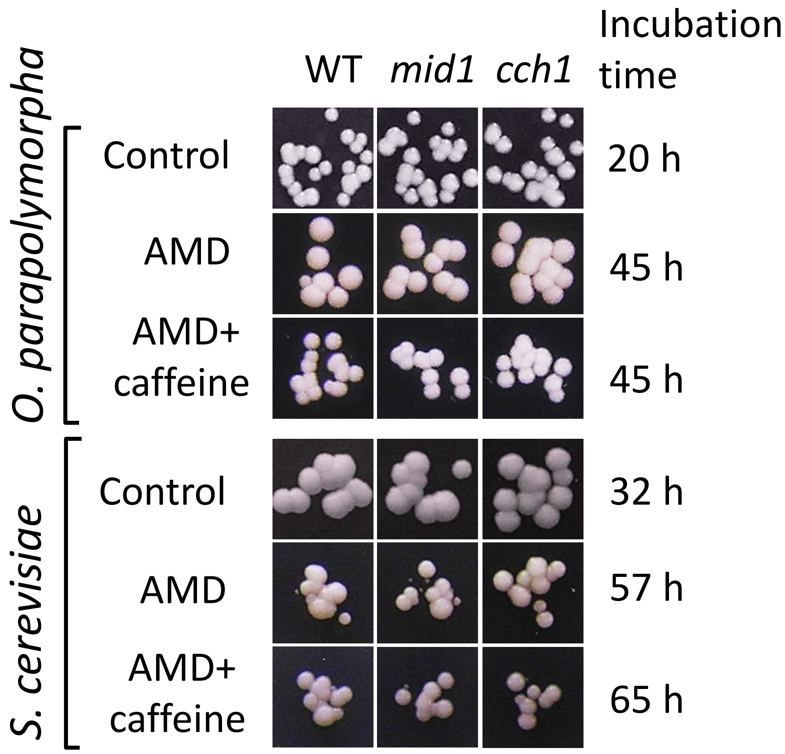

Figure S3. Growth of yeast colonies on YPD (control), YPD supplemented with 1 mM amiodarone (AMD) or 1 mM amiodarone and 2.5 mM caffeine (AMD + caffeine). *O. parapolymorpha* and *S. cerevisiae* plates were incubated at 37°C or 30°C, respectively.

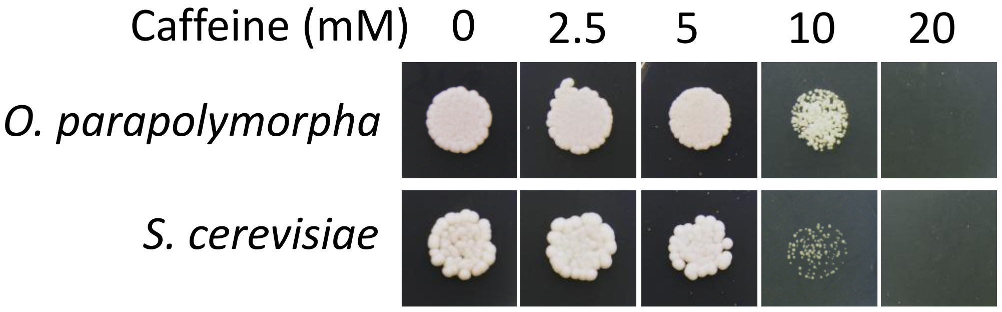

Figure S4. Ability of yeast cells to grow in presence of different caffeine concentrations. Plates were incubated 2 days at 30°C.

Table S1. PCR primers

Primer	5'-3' Sequence
OpMID1L1	CTTCGTCGCTGCTCCGCCAA
OpMID1U1	GCAACCTCATTCAGATCACC
OpMID1AU1	GCGGAAGAGGCTGGGCTGAA
OpMID1AL2	GGGGCGTGCTGTTGGCTCGT
oriA	CCTATGGAAAAACGCCAGCAA
SL3	GGTGATGCTGTCGCCGAAGAAG
